# Current management and future challenges in salivary glands cancer

**DOI:** 10.3389/fonc.2023.1264287

**Published:** 2023-09-19

**Authors:** Laura D. Locati, Renata Ferrarotto, Lisa Licitra, Marco Benazzo, Lorenzo Preda, Davide Farina, Gemma Gatta, Davide Lombardi, Piero Nicolai, Vincent Vander Poorten, Melvin Lee Kiang Chua, Barbara Vischioni, Giuseppe Sanguineti, Patrizia Morbini, Isabel Fonseca, Davide Sozzi, Anna Merlotti, Ester Orlandi

**Affiliations:** ^1^ Department of Internal Medicine and Therapeutics, University of Pavia, Pavia, Italy; ^2^ Medical Oncology Unit, Istituti Clinici Scientifici Maugeri Istituto di Ricovero e Cura a Carattere Scientifico (IRCCS), Pavia, Italy; ^3^ Department of Thoracic and Head and Neck Medical Oncology, The University of Texas MD Anderson Cancer Center, Houston, TX, United States; ^4^ Head and Neck Medical Oncology Unit, Fondazione Istituto di Ricovero e Cura a Carattere Scientifico (IRCCS) National Cancer Institute, Milano, Italy; ^5^ University of Milan, Milano, Italy; ^6^ Department of Clinical, Surgical, Diagnostic, and Pediatric Sciences, University of Pavia, Pavia, Italy; ^7^ Department of Otorhinolaryngology, Fondazione Istituto di Ricovero e Cura a Carattere Scientifico (IRCCS) Policlinico San Matteo, Pavia, Italy; ^8^ Diagnostic Imaging and Radiotherapy Unit, Department of Clinical, Surgical, Diagnostic, and Pediatric Sciences, University of Pavia, Pavia, Italy; ^9^ Radiology Institute, Fondazione Istituto di Ricovero e Cura a Carattere Scientifico (IRCCS) Policlinico San Matteo, Pavia, Italy; ^10^ Azienda Socio-Sanitaria Territoriale (ASST) Spedali Civili di Brescia, Division of Radiology and Department of Medical and Surgical Specialties, Radiological Sciences and Public Health, University of Brescia, Brescia, Italy; ^11^ Evaluative Epidemiology Unit, Fondazione Istituto di Ricovero e Cura a Carattere Scientifico (IRCCS) National Cancer Institute, Milano, Italy; ^12^ Department of Otorhinolaryngology - Head and Neck Surgery, University of Study, Brescia, Italy; ^13^ Unit of Otorhinolaryngology - Head and Neck Surgery, University of Study, Padova, Italy; ^14^ Otorhinolaryngology-Head and Neck Surgery, Leuven Cancer Institute, University Hospital of Leuven, Leuven, Belgium; ^15^ Department of Oncology, Section Head and Neck Oncology, Katholieke Universiteit Leuven, Leuven, Belgium; ^16^ Division of Radiation Oncology, National Cancer Centre Singapore, Singapore, Singapore; ^17^ Radiation Oncology Clinical Department, National Center for Oncological Hadrontherapy, Pavia, Italy; ^18^ Department of Radiotherapy, Istituto di Ricovero e Cura a Carattere Scientifico (IRCCS) Regina Elena National Cancer Institute, Roma, Italy; ^19^ Unit of Pathology, Department of Molecular Medicine, University of Pavia, Foundation Istituto di Ricovero e Cura a Carattere Scientifico (IRCCS) Policlinico San Matteo, Pavia, Italy; ^20^ Anatomia Patológica, Instituto Português de Oncologia Francisco Gentil, University of Lisbon, Lisbon, Portugal; ^21^ Department of Medicine and Surgery, School of Medicine University of Milano-Bicocca, Monza, Italy; ^22^ Maxillofacial Surgery Unit, Fondazione Istituto di Ricovero e Cura a Carattere Scientifico (IRCCS) San Gerardo dei Tintori, Monza, Italy; ^23^ Department of Radiation Oncology, Santa Croce and Carle Teaching Hospital, Cuneo, Italy

**Keywords:** salivary gland cancer, rare cancer, surgery, heavy particles, targeted therapy

## Abstract

Salivary gland cancers (SGCs) are rare, accounting for less than 5% of all malignancies of the head and neck region, and are morphologically heterogeneous. The diagnosis is mainly based on histology, with the complementary aid of molecular profiling, which is helpful in recognizing some poorly differentiated, borderline, or atypical lesions. Instrumental imaging defines the diagnosis, representing a remarkable tool in the treatment plan. Ultrasound and magnetic resonance are the most common procedures used to describe the primary tumour. The treatment of SGCs is multimodal and consists of surgery, radiotherapy, and systemic therapy; each treatment plan is, however, featured on the patient and disease’s characteristics. On 24 June 2022, in the meeting “Current management and future challenges in salivary gland cancers” many experts in this field discussed the state of the art of SGCs research, the future challenges and developments. After the meeting, the same pool of experts maintained close contact to keep these data further updated in the conference proceedings presented here. This review collects the insights and suggestions that emerged from the discussion during and after the meeting per se.

## 1 Introduction

On June 24, 2022, a one-day meeting entitled “Current management and future challenges in salivary glands cancer” took place at CNAO (Italian National site for Hadrontherapy) in Pavia, Italy. Several international experts in the field have been involved to bring their experience on the management and the research in salivary gland cancers (SGCs). A multidisciplinary overview contributed to turn on the light on these challenging tumours, especially regarding the future research and development. In this review, we describe the current landscape in SGC treatment, focusing on the novelties in diagnosis, surgery, radiotherapy, and systemic therapies that emerged during the meeting.

## 2 Epidemiological update

SGCs are rare, accounting for less than 5% of all malignancies of the head and neck (HN) region. The WHO Global Cancer Observatory reported 53.583 new diagnoses in 2020 worldwide; the incidence was 0.59 and the mortality 0.23 per 100,000/year (1). Across all European countries, the Eurocare register, which collects data on rare tumours including SGCs, reports an incidence of 0.91 per 100,000/year for malignant epithelial tumours of major salivary glands and 0.43 per 100,000/year for salivary gland-type tumours of the minor salivary glands (2).

The incidence is stable over time, without increment in the risk, except for the elderly population. In 2020, 43% of SGCs occurred in the elderly, causing 12,339 cancer-specific deaths, with a male-to-female ratio of 1.3:1. In the next two decades, the new diagnoses in the elderly age group are expected to account for 80% of the total SGCs diagnoses (3). The review of the Surveillance, Epidemiology and End Results (SEER) Program database indicated that the incidence of major salivary glands and salivary gland-type cancers in patients over 65 years was 4 and 7 times higher, respectively, than that reported in younger patients and the overall 5-year survival rates were significantly better in young than in elderly subjects who, more frequently, presented histotypes with poor prognosis (e.g. salivary duct cancer) or unspecified histotypes (3). Indeed, almost half of SGCs cases from the SEER dataset presented at diagnosis with localized disease, without significant differences between major salivary glands and salivary gland-type carcinomas, while elderly patients were diagnosed more frequently at a metastatic stage (3).

## 3 Pathological classification and molecular characterization

The diagnosis of SGCs may be challenging to the pathologist because it is a morphologically heterogenous group of neoplasms. The characteristics of each neoplasm have been specified in the updated SGCs classification which the World Health Organization (WHO) has recently released (4). The most important novelties are i) the introduction of molecular data to define new entities; ii) the attention to cytological findings according to the Milan System; iii) the attention to high-grade transformation which may determine a negative prognosis (5). Many types of SGCs (e.g. mucoepidermoid carcinoma, adenoid cystic carcinoma, acinic cell carcinoma, secretory carcinoma, polymorphous adenocarcinoma, hyalinizing clear cell carcinoma, mucinous adenocarcinoma, and microsecretory adenocarcinoma) are defined according to the presence of recurrent genomic alterations, such as gene fusions and tightly tumour-type specific mutations (**Table 1**).

**Table 1:** Salivary gland cancer, according to the presence of recurrent genomic alterations (6, 7).

Histotype	Molecular alterations	Fusion (major)	Fusion (alternative)
Adenoid cystic carcinoma	*NOTCH1* mutation; *EGFR* and *KIT* overexpression	*MYB-NFIB*	*MYBL1-NFIB*
Mucoepidermoid carcinoma	*PI3KCA, POU6F2, BRCA1/2; CDKN2A* mutation	*CRTC1-MAML2*	*CRTC3-MAML2*
Acinic cell carcinoma	*CDKN2A* and *PPP1R13B* deletion	*NR4A3*	*HTN3-MSANTD3*
Secretory carcinoma	*PRSS1, MLH1, MUTYH*, and *STK11* mutation	*ETV6-NTRK3*	*ETV6-RET; ETV6-MET*
Hyalinising clear cell carcinoma	Not reported	*EWSR1-ATF1*	*EWSR1-CREM;* *EWSR1-CREB1*
Intraductal carcinoma	*KRAS* and/or *PI3KCA* mutations	*NCOA4-RET*	*TRIM27-RET*
Carcinoma ex pleomorphic adenoma	HER-2 overexpression and *ERBB2* amplification; *HRAS* mutation; *PI3KCA* mutations; *PTEN* loss	*PLAG1-HMGA2*, *NFIB-PLAG1*	*CTNNB1, LIFR, FGFR1*
Salivary duct carcinoma	AR overexpression; HER-2 overexpression and *ERBB2* amplification; *BRAF*, *HRAS, PI3KCA, EGFR* and *NF1* mutation; *PTEN* loss	*PLAG1-CTNNB1*	
Myoepithelial carcinoma	*KRAS* and *HRAS* mutation; *SMARCB1* deletion	*TGFBR3-PLAG1*	*PLAG1; HMGA2; EWSR1*

Recurrent gene defects become, therefore, valuable and helpful for use in diagnostically challenging cases, not only for examining poorly differentiated lesions but also for recognizing borderline or atypical lesions. The next-generation sequencing approach may contribute to clarifying some heterogeneous groups, such as adenocarcinoma, not otherwise specified (NOS) (8). However, most entities are defined based on histology and immunohistochemistry findings, and molecular characterization is not mandatory for the diagnosis (9). However, molecular diagnosis can be supplementary in terms of providing information on biological behaviour, as well as, the suitability of a patient to targeted therapies. Indeed, some gene defects can help to identify some potential targets for therapy; currently, the predictive role of molecular alterations is still not relevant, except for the *RET* and *NTRK* genes translocation that can be targeted by specific inhibitors (e.g. selpercatinib or pralsetinib for *RET* and entrectinib or larotrectinib for *NTRK*) (10, 11); androgen receptor overexpression in salivary duct carcinoma handled with androgen deprivation therapy (12, 13), and HER2 overexpression/amplification treated with trastuzumab, since other HER2 targeted agents available perhaps generalize to HER2 targeted therapies (14).

Another aspect of novelty in the new classification is the attention to the high-grade transformation/dedifferentiation. High-grade transformation is associated with aggressive clinical behaviour and poor prognosis, regardless of the background histotypes. Adenoid cystic adenocarcinoma (ACC) more frequently undergoes high-grade transformation, usually *de novo* at the initial presentation and, more rarely, at recurrence. The presence of high-grade transformation may be detrimental to morphological diagnosis because of the partial or total loss of distinct morphology of background histotype; in this case, molecular features may be useful to characterize the tumour. The genetic bases which determine the shift toward high-grade transformation have been not completely elucidated yet (15).

Some unresolved issues still exist in the WHO Classification 2022: for example, the definition of mucinous adenocarcinomas or to classify the oncocytic carcinoma no more as an independent entity.

The classification of WHO 2022 reserves an important role to cytology in the diagnosis, introducing the Milan System for reporting. The Milan System provides a very practical SGC classification from a cytological point of view (**Table 2**).

**Table 2:** Diagnostic categories in the Milan System for Reporting Salivary Gland Cytopathology (16).

Diagnostic category	
I. Non diagnostic
II. Non-neoplastic
III. Atypia of Undetermined Significance (AUS)
IV. Neoplasm
IVA. Neoplasm: benign
IVB. Neoplasm: salivary gland neoplasm of uncertain malignant potential (SUMP)
V. Suspicious for Malignancy
VI. Malignant

Diagnostic category correlates with the risk of malignancy (ROM), tier I ROM 25%, tier II ROM 10%, tier III ROM 20%, tier IVA ROM < 5%, tier IVB ROM 35%, tier V ROM 60%, tier VI ROM 90%.

## 4 Radiological diagnosis of malignant tumours from both major and minor salivary glands

Imaging plays essential role in treatment planning, in terms of tumour characterization and of locoregional spread detection. Ultrasound (US) imaging with high frequency is the first examination and can be considered conclusive, in case of small lesions or clinically defined and/or confined to the superficial lobe of the parotid gland. In most cases, US can distinguish between benign and malignant tumours, as benign lesions present regular and well-defined margins, a homogeneous hypoechoic structure, and demarcated vessel distribution, while malignant tumours are poorly defined with an irregular shape, blurred margin, and hypoechoic, heterogenous internal architecture and perfusion. However, in some cases as in lower-grade lesions, benign and malignant salivary gland tumours may have a similar US patterns. They appear well-defined and may display a lobulated border and homogeneous internal architecture, as well as, pleomorphic adenomas may have an irregular shape with heterogeneous echo structures. Similarly, about 60% of benign and 50% of malignant tumours are poorly vascularized, while all Warthin tumours, 15% of pleomorphic adenomas, and 38.8% of malignant tumours are well-vascularized (17). Thus, although US is a sensitive and specific technique, about 18-20% of specimens remain non-diagnostic and indeterminate (18).

US is frequently used to perform biological sampling and fine-needle aspirate cytology (FNAC), as in experienced hands is inexpensive, easy to perform, well-tolerated, and safe. Core-needle biopsy (CNB) has a higher sensitivity in diagnosing malignant neoplasms and allows tumour characterization and grading in most of the cases. The technique is less operator-dependent and has a lower rate of indeterminate and non-diagnostic specimens (19). It is, however, more cumbersome with some potential complications and that is why, in the recent ESMO/EURACAN guidelines, a stepped approach is recommended, performing CNB in patients where FNAC is inconclusive (20).

Besides US, magnetic resonance (MRI) represents the imaging technique of choice as it can provide both a morphological and structural analysis by combining conventional and functional sequences. It is valuable to characterize lesions especially when clinical and US evaluations are doubtful or cyto-histological sampling is not conclusive or difficult to perform MRI may have a role in surgical planning in presence of symptoms suggestive of malignancy (such as pain, paralysis of the VII nerve, and lymphadenopathies) and in case of large lesions or lesions involving the deep lobe of the parotid gland (21, 22).

Functional MRI procedures contribute to better discrimination and describe specific types of SGCs. Diffusion-weighted (DWI)-MRI sequence and dynamic contrast-enhanced (DCE) can differentiate pleomorphic adenoma from Warthin tumours. In DCE, the degree of tumour enhancement is plotted against time and the acquired signal generates a time-intensity curve; four time-intensity curves have been characterized. Most pleomorphic adenomas have a type A curve (time to peak was more than 120 seconds), while almost all Warthin tumours have a type B curve (time to peak was 120 seconds or less with high washout ratio, ≥30%); most malignant tumours are characterized by a type C curve (time to peak was 120 seconds or less with low washout ratio, <30%), which is a criterion for predicting salivary gland malignancy with 79% sensitivity and 95% specificity (23). MRI also allows an investigation of the relationship between facial nerves and tumoural mass and the presence of perineural spread. Therefore, the combination of conventional MR and functional imaging contributes to better defining the tumour and helps in treatment planning (21) (**Table 3**).

**Table 3:** Conventional MRI features in pleomorphic adenoma, Warthin tumour, and malignant tumour (24, 25).

Type of lesion	Typical MR Characteristics	Contrast-enhancement pattern	Additional characteristics
**Pleomorphic adenoma**	Low signal on T1w, bright signal on T2w Well-defined, lobulated margins with hypointense capsule	Marked heterogeneous nodular enhancement	Cellular subtypes or with fibrosis content associated with low T2 signal
**Warthin tumour**	Cystic portions with high signal on unenhanced T1w and intermediate-to-high signal on T2w	Solid portions with low-intermediate enhancement	Located in the inferior portion of the parotid gland, often bilateral
**Malignant tumours**	low signal on T2w (high cellularity). Undefined borders, invasion of surrounding structures. Macroscopic perineural spread. Lymphadenopathies	Homogeneous or heterogeneous contrast-enhancement. Necrosis and cystic changes are not specific features	Low-grade lesions may have MRI features comparable to benign lesions (homogeneous signal intensity, well defined borders and capsule-like rim enhancement)

MRI, magnetic resonance imaging T1w, T1 weighted; T2w, T2 weighted.

Computed Tomography (CT) represents an alternative when MRI is contraindicated or represents an additional exam in cases of suspected involvement of bony structures. CT is less sensitive than MRI in detecting the presence of perineural diffusion, in such cases it may demonstrate enlargement or erosion of skull base foramina (17).

In presence of histologically confirmed malignant lesions, especially if high grade, CT examination can be extended to thorax and abdomen for staging purpose (17).

Some differences can be accounted for major and minor salivary gland imaging. First of all, the anatomy of the minor salivary gland is different, as minor glands are located especially in the oral cavity (mostly lips, posterior 1/3 of the hard palate, base of the tongue), in oro- and nasopharynx, paranasal sinuses, larynx, and trachea, with a submucosal location. The anatomic site of the primary tumour influences the choice of the imaging acquisition protocol. In the differential diagnosis of minor SGCs, it should be noted that the rate of malignant tumours is higher than in major SGCs, with pleomorphic adenoma, mucoepidermoid carcinoma, and ACC being the most common histological type (**Table 4**). Other histotypes have sites and patterns overlapping; therefore, imaging is not enough to give a diagnosis in most cases.

**Table 4:** Clinical and radiological features of the most common tumours in minor salivary glands.

Histotype	Pleomorphic adenoma	Mucoepidermoid carcinoma	Adenoid cystic carcinoma
**Radiological pattern**	Usual MRI-CT pattern DWI -	Imaging findings variable, depending on grade	DWI + Permeative growth pattern
**Anatomic site**	Hard palate, upper lip	Palate	Paranasal sinus (30%), oral cavity
**Typical features**	Rarely malignant	Children and young adults	Perineural spread

MRI, magnetic resonance imaging; CT, computerized tomography; DWI, diffusion-weighted magnetic resonance imaging.

The differential diagnosis should encompass other entities, namely “tumour mimickers”, including mucocele, IgG4-related disease (26), and necrotizing sialometaplasia. Local invasion and a permeative growth pattern are features of ACC, with the involvement of bone, and soft tissue. Perineural spread of minor SGCs involves maxillary (tumours arising from hard palate and spheno-palatine area) and mandibular nerves (tumours arising from nasopharynx), VII cranial nerves, mostly via interconnections with V cranial nerves, and lower cranial nerves (tumour arising from nasopharynx extending to the skull base). All these characteristics of the tumours of minor salivary glands reflect on imaging protocols in terms of field-of-view and spatial resolution. The field of view should be adapted to the site that should be visualized and the resolution should be maximized to identify all lesions.

## 5 Surgery of salivary glands cancers: current status and recent advances

Surgery is often the upfront treatment for all SGCs; however, the most important prognostic factors (e.g. high-grade lesion, high pT-category, perineural spread, especially if microscopic) that may affect the outcomes are not often available before surgery. The Vander Poorten Scoring System may help in estimating prognosis and decision making. This index allows for a weighted estimate of an individual patient’s prognosis in both the pre- and the post-operative situation (27, 28), and by now has been repeatedly externally validated (29–33) and it is also available as a nomogram (34).

The main principles of surgery are complete resection (R0) with adequate margins and the preservation (or restoration) of vital function. Regarding the definition of “adequate margins”, no relevant differences have been observed between negative and close (< 5 mm) margins for all SGC sites (35), except for the oral cavity. For ACC, there is consensus that a close margin (R1) resection makes sense in enhancing local control in combination with postoperative radiotherapy as compared to using radiotherapy alone (35, 36). In this respect, patients with an expected R2 margin are preferably sent for primary radiotherapy, and heavy ion therapy is preferred in this situation (20).

The vast majority of high-grade tumours require a combination of surgery and radiotherapy, although the most efficacious technique for radiotherapy delivery e.g., photons versus protons versus carbon ion therapy remains undefined. In the adjuvant setting, the use of platinum-based chemoradiotherapy was well tolerated but up to now did not demonstrate any survival improvement, compared to radiotherapy alone in patients with high-risk salivary gland cancers (37). Indeed, the use of concomitant chemoradiation in adjuvant setting is discouraged by the recent guidelines (20, 38), outside of clinical trials; the RTOG-1008 trial (NCT01220583) has completed recruitment, and currently, we await the first results.

### 5.1 Surgery for MiSGC

Minor SGCs are rare and heterogeneous in terms of histology, grade, and site of origin. In these tumours, negative prognostic factors are advanced stage at presentation including advanced T classification, positive margins (R1), positive nodes, perineural and – especially for ACC – intraneural spread (39), sinonasal and nasopharyngeal site of origin, lymph node ratio, and high-grade. Survival is quite good, but high-grade tumours are associated with a dismal prognosis (40). The surgical management of minor SGCs is particularly challenging and should be centralized in centres with large experience. Transoral surgery, transnasal endoscopic surgery, and combined transoral-transnasal techniques are becoming common surgical procedures to manage minor SGCs of oropharyngeal or sinonasal origin (41). Since the past 2 decades, transoral surgery is traditionally performed using laser microscopic surgery, through laryngoscopes and oropharyngoscopes, but the surgeon is limited by the line of sight through a positioned scope, while only a tangential cutting plane can be used. Frequent repositioning of scopes results in a piecemeal resection, and the technique needs a demanding learning curve. In specific indications and given good exposure, the Da Vinci ^®^ robot for transoral surgery can result in improved maneuverability and visualization, thus overcoming the limits of transoral laser microsurgery and giving access to selected tumours that are otherwise hard to approach. The underlying idea is to use a minimally invasive natural orifice surgery, reducing the interference with surrounding tissues when compared to traditional transcervical/transmandibular approaches. Current evidence in the use of this technique in minor SGCs concerns tumours located in the oropharynx at the base of the tongue and, very rarely, supraglottic locations; limited evidence is available on the advantages of robotic surgery for parapharyngeal SGCs of minor and major salivary gland origin (42, 43). Based on the experience with squamous cell carcinomas (SCC), it is suggested that, in selected patients, transoral surgery can result in a shorter recovery and hospital stay and better functional outcomes than open approaches; in selected patients with SCC in the salvage setting, the transoral approach also shows functional and oncological superiority (44). We should, however, remain careful in extrapolating this experience with SCC to SGCS of minor salivary gland origin, the latter having a known tendency to submucosal spread and perineural invasion, complicating a good resection. To date, the data supporting this approach in SGCs remain limited and often related to small case series and retrospective studies with potential inclusion bias (42, 43). Nevertheless, transoral robotic surgery, followed by adjuvant radiotherapy, should be considered a valuable option in the multidisciplinary management of minor SGCs, achieving durable disease-free survival in well-selected patients (43). In the same line, endoscopic endonasal resection can be applied in the nasopharynx (in SGCs without involvement of the internal carotid artery, and with minimal skull base extension) and in the sinonasal tract and skull base (45). The combination of endoscopic and transoral approaches, e.g. in the endoscopic-assisted maxillectomy, has the dual advantage of better delineating the posterior margin by drilling the pterygomaxillary junction while avoiding facial incisions (46).

For ACC located in the nasopharynx and in the sinonasal tract, the main issue is the nerve invasion, not only limited to perineural invasion and inflammation but also including intraneural invasion, which resulted as an independent predictor of poor prognosis (39). In particular, definitive upfront radiotherapy should be considered for ACC of the nasopharynx.

### 5.2 Surgery for Major SGCs

The surgery of major SGCs consists in most of the cases in superficial parotidectomy or total parotidectomy, to obtain a complete resection with adequate free surgical margins (47). The extent of resection performed may differ according to the local extension and specific growth pattern of the tumour (48). The debate on parotidectomy is still open: a superficial parotidectomy can be sufficient and adequate for superficial lobe lesions and in presence of normal mobility and functioning of the VII nerve, while total conservative parotidectomy is preferred when deep lobe parotid lymph nodes are at risk or involved (49). Both ASCO and ESMO/EURACAN guidelines (20, 38) suggest that at least parotidectomy with the removal of additional parotid tissue should be recommended for advanced or high-grade cancer, if it is deemed to not place the facial nerve at significant risk, but the latter is obviously related to the experience of the surgeon. Prophylactic deep lobe parotidectomy may be indicated with high-grade tumours and in presence of lymph node involvement, especially using the en bloc technique that limits the risk to facial nerve damage (50). Total parotidectomy for small malignant tumours is not supported by significant evidence as no randomized clinical trials are available and the local recurrence rate is very low in the early stage, if adequately treated with postoperative radiotherapy (51–53). Nevertheless, current guidelines promote total parotidectomy in tumours with pre-operative known type and high-grade (20, 38). Total parotidectomy is indicated for a tumour in the deep lobe, retromandibular area, upper part of the stylomandibular tunnel, and in presence of obvious malignant tumours with extraparenchymal extension or neck metastasis. Mandibulotomy should be considered (but surely is not always needed) for deep lobe malignant tumours, or parapharyngeally recurring pleomorphic adenomas. Reconstructive surgery is aimed at minimizing aesthetic deformity and maximizing the functionality in radical parotidectomy with VII nerve sacrifice. Classical combinations are static reconstruction, free fasciocutaneous or muscle flaps for soft tissue and skin replacement, and free nerve cable grafting to restore the sacrificed facial nerve; new developments resulting in better and quicker recovery are the use of vascularized nerve grafts and of the masseteric nerve transfer (41).

## 6 Radiotherapy: when, how, and where

According to ASCO and ESMO/EURACAN Guidelines (20, 38), postoperative radiotherapy should be offered to all patients with ACC, and for the other SGC types for high-grade tumours, positive margins, perineural invasion, lymphovascular invasion, lymph node metastases, and pT3-4 tumours; it should also be considered an option for patients with close margins or intermediate-grade tumours (38). Radiotherapy should be suggested also to patients who are not eligible for surgical resection because of the extent of the disease or in case of anticipated R2 resection or the presence of clinical comorbidities. Elective nodal irradiation is indicated for a selected group of patients with high-grade tumours or advanced T status that did not undergo neck dissection at the time of the primary resection.

The scenario is different for low-grade SGCs (e.g. low-grade mucoepidermoid carcinoma, classical acinic cell carcinoma, myoepithelial carcinoma, all polymorphous adenocarcinomas). No data from randomized studies are currently available on the role of radiotherapy in low-grade SGCs and recommendations mainly derive from retrospective studies and expert opinions. The treatment paradigm of a malignant low-grade tumour consists of surgical resection in all salivary sites, followed by postoperative radiotherapy in presence of the risk factors mentioned above or in case of recurrence and it should be accounted for that almost 50% of low-grade SGCs (54) and almost 30% of ACC have at least one high-risk feature (55). On the other hand, radiotherapy is not suggested in pT1 and pT2 low-grade cancers without additional negative prognostic factors; indeed, in a study cohort on more than 800 patients with surgically treated SGCs, the use of post-operative radiotherapy did not change the survival rates in the subset of patients with stage I/II, close margins (< 1 mm) and low- or intermediate-risk histologic type (56). But one should remain cautious in that there is still inevitable selection bias in this institutional cohort, even if there is correction for confounding via multivariate analysis.

For ACC, the standard treatment consists of radical surgery and postoperative radiotherapy, especially for locally advanced disease and in presence of the risk factors mentioned above. Due to its well-known radioresistance, ACC remains a major challenge for radiation oncologists. Its horseshoe shape often embraces or intersects radiosensitive structures following neural pathways: indeed, high conformational radiotherapy techniques are required to reduce the dose delivered to normal structures avoiding radiation induced severe toxicities. In this regard, particles, including protons, neutrons and carbon ions, with different physical features appear to reduce the low-to-moderate dose of photon radiotherapy (RT) by inverting the depth dose profile of energy deposition through the matter. In contrast to photons, the dose at the beam entrance is relatively lower than in the Bragg peak, where most energy is deposited in a limited depth interval with a consequently reduced irradiation of healthy tissues along the beam path. In addition, neutrons and carbon ion radiation therapy (CIRT) show several advantages compared to photons. In particular carbon ions have a superior relative biological effectiveness (RBE) that is estimated at least a 2–3-fold factor in comparison to photons and protons. Neutrons and carbon ions entered the clinical practice from some decades. Good local control (LC) rates from early neutron studies on SGCs, including the pivotal phase III trial conducted by Radiation Therapy Oncology Group (RTOG) and Medical Research Council (MRC) in the 1980’s were unfortunately reached at the expense of considerably higher late toxicities compared to photons (57). Thus, it led to the investigation of CIRT therapy for these tumours, ACC, particularly when surgery is not an option. In addition to the dosimetric advantages with steep dose gradients beyond the Bragg peak, steering of carbon ions is much more convenient than for neutrons. The interest in carbon ion arose in Germany (58) and Japan (59, 60) and rapidly spread around the world, with many particle facilities that are built even in Europe and China. Evidence that ACC may benefit from CIRT, alone or in combination with photons based intensity modulated RT in terms of LC, overall survival (OS) and toxicity, including R2 and inoperable cases has been reported in the latest years (61–64). Especially in Akbaba et al. for paranasal sinuses after CIRT boost it was reported higher toxicity rate (>G3) in the postoperative intensitiy modulated radiotherapy (IMRT) + CIRT cases in comparison to the primary IMRT + CIRT, with comparable results in terms of LC (58). In a series of 184 patients with ACC of the head and neck treated with CIRT at CNAO from January 2013 to June 2020 worse OS was reported for patients with any gross tumour volume (GTV) at pre-CIRT MRI compared to macroscopically resected patients (p=0.008), with shorter OS in patients after debulking surgery and unresected patients (43% and 54% 5 years OS) compared to R1 postoperative patients with macroscopic disease at pre CIRT MRI (78% OS) and patients with microscopic disease (93%, p=0.014).

It is difficult to exhaustively delineate the toxicity scenario of CIRT as different prescription doses, different biological models for dose prescriptions, and different dose constraints are used in each centre. In addition, some discrepancies are observed in the way to evaluate the impact of therapy, especially for example on brain toxicity. Consensus initiatives are necessary to standardize as much as possible treatments with CIRT and the evaluations of toxicity during the follow-up as it has been proposed for proton therapy (65). Proton therapy can be used to achieve a good dose distribution in complex ACC volumes and may be potentially advantageous over advanced photon techniques in selected cases and for children and young adults to reduce low dose splash of conventional photon RT. High local control was achieved in a Japanese series of 25 patients (3-year LC 63%) (66) and in another American series of 19 patients (2-year OS 93) (67) treated with radical proton therapy. An excellent outcome (5-year LC 93%) was reported in a French series of 23 patients treated with mixed photon/proton beams when post-surgical flap insertion is performed or in young patients (68). It is important to remember that each tumour localization and histology needs a specific approach. In paranasal sinuses and palate, the most common histological subtype is squamous cell carcinoma, and rarer variants are olfactory neuroblastoma, adenocarcinoma, mucoepidermoid carcinoma, ACC, undifferentiated sinonasal carcinoma, neuroendocrine carcinoma, and chordoma. Postoperative radiotherapy is indicated almost in all patients and treatment recommendations are agnostic to histological subtypes. However, some attention should be paid in presence of a positive margin, in proximity to crucial structures, and according to the status of the reconstructed flap and irradiation of the neck.

Some issues of radiotherapy are still present in paranasal sinuses, although many advances have been done in this field in the last years (69). The tumour clinical target volume dosimetry is challenging as the dose is often limited to respect the constraints of critical structures and is particularly critical in unresectable diseases. In some cases, excessive doses with hotspots >10% of the prescribed dose to the skull base, skin, and flap can occur; it should be cautious to avoid exceeding doses within critical structures such as optic nerve and chiasm or to cause other equally debilitating complications including flap necrosis, ocular infections, eating difficulty as all these side effects can compromise short- and long-term quality of life (70). Finally, the anatomical volume to be included in case of perineural invasion remains controversial, in particular no consensus exists to treat electively the skull base only when a named nerve is involved or include it routinely even when a microscopic perineural inflammation is reported.

## 7 Neck involvement in salivary gland cancer

The treatment of salivary gland cancer with clinically negative lymph nodes is still unclear. In this patients category we need to look at the presence of the risk factors for occult neck disease and deciding to treat the neck when the probability, based on the combined presence of different risk factors, exceeds the threshold of 15–20%. Risk factors predicting micrometastases are clinical characteristics, such as age (>54 years), pain, facial nerve dysfunction and stage >T2, and pathological as intermediate- or high-grade tumour, extraglandular soft tissue invasion and lymphatic invasion (71). A different distribution of occult metastases in the neck in cN+ and cN0 has been reported (72). The rate of occult nodal disease ranges from 10.2 and 22.4% in patients with cN0 parotid cancer (73) and from 10 to 40% in patients with cN+ parotid cancer (48). According to the ESMO/EURACAN/EURACAN and NCCN guidelines, management of cN0 can be different according to the primary site and the presence of high-risk features. Elective neck dissection is suggested in case of major salivary gland cancer in presence of “high-grade and/or T3–4 tumours” (20, 74). Elective RT could be a second option in high-risk patients that end up in this category depending on definitive histopathology of the resected primary (71). In case of primary from minor salivary glands of the head and neck or sublingual gland, elective neck dissection is always recommended (20, 74). In patients with cN+ all levels of the neck are involved, as well as intra and peri-parotid nodes (72, 75, 76). Considering the classical TNM of squamous cell carcinoma of the upper airways, SGCs have a peculiar biology as no contralateral nodal involvement is described, rarely metastases measure more than 6 cm in diameter, and the role of extra-nodal extension is at least debatable. This biology reflects on a different impact of nodal involvement: the quantitative burden of nodal disease is an important determinant with a progressive impact on prognosis, while extranodal extension does not seem to impact on prognosis (77). Intraparotid nodal involvement is another negative prognostic factor that should be included in treatment planning (78). The inclusion of the intraparotid lymph node status into the lymph node assessment with the log odds of pN+ led to robust prognostication, regardless of the T status (79). Therefore, the intraparotid node should be assessed after surgery in every single case and, definitively, a novel N staging system tailored to major salivary glands should be evaluated.

## 8 Systemic therapies for recurrent/metastatic salivary gland cancers

Systemic therapies for recurrent/metastatic SGCs are chemotherapy and targeted therapy for ACC and chemotherapy, targeted therapy, hormonal therapy, and immunotherapy for non-ACC. It is not clear which is the best therapeutic approach because randomized trials are lacking. Chemotherapy provides a low response rate in ACC (5-22%), while in other histotypes the response rate ranges from 30 to 40%. However, the effect on overall survival has not been demonstrated yet, but there is a potential impact on quality of life (80). Chemotherapy is generally reserved for palliative care for an advanced disease that is not manageable with local therapies such as surgery and/or radiation (80). ACC is a biphasic tumour consisting of myoepithelial and epithelial cells, with *MYB/MYB L1-NFIB* rearrangements which occur in almost 65% of cases. The rate of distant failure after curative treatment ranges from 40 to 50%, and approximately 15% of cases have an aggressive disease course. No standard of care system therapy for patients with metastatic disease is currently available, and chemotherapy, anti-angiogenic agents, and checkpoint inhibitors have limited activity. Noteworthy, despite the biological variability of the disease, all patients are treated in the same way. The aggressiveness of ACC depends on its molecular profile and, in particular, on the mutational status of the *NOTCH 1* gene. About 15% of patients show mutations in *NOTCH 1* gene, most of them are located in the negative regulation PEST domain. *NOTCH*-mutated patients have a peculiar phenotype with a solid component, bone and liver metastases, and advanced stage IV (81); *NOTCH 1* mutations have been associated with a worse prognosis (82).

In proteogenomic studies, consensus clustering identified two distinct ACC subtypes, ACC-I (37%) and ACC-II (63%). ACC-I had strong upregulation of *MYC*, *MYC* target genes, and mRNA splicing, enrichment of NOTCH-activating mutations, and dramatically worse prognosis. ACC-II exhibited upregulation of TP63 and receptor tyrosine kinases (AXL, MET, and EGFR) and a less aggressive clinical course. TP63 and MYC were sufficient to assign tumours to ACC subtypes, which was validated in one independent cohort by IHC and two additional independent cohorts by RNA-sequencing (83).

The presence of multiple targetable protein/pathways alterations in each ACC subtype provides opportunities for combination therapy for this disease (83). Potential drug targets in ACC-I are PRMT5, NOTCH 1 (84), and BCL2, while in ACC-II these are EGFR, AXL, and MEK/AKT pathways (83). Xenograft models of ACC-I were responsive to PRMT5 inhibition with a block of tumour growth (85); AL101 determined tumour regression in *NOTCH* activated ACC-I models and further synergic activity was observed when used in combination with BCL2 inhibitor or palbociclib (86). The ACCURACY study investigated the response to the NOTCH inhibitor AL101 in patients with recurrent and/or metastatic ACC harbouring *NOTCH* activating mutations (87), while the response rate was overall low (8.3 to 14.6%), the disease control rate was 66.7-70.7% and clinical benefit was noted in a proportion of patients. To better understand the biologic changes induced by pharmacologic NOTCH inhibition in NOTCH-mutated ACC and guide rationale combinatorial therapy, a window of opportunity trial is currently being conducted with the gamma-secretase inhibitor AL101. AXL is another promising target for therapy; in preclinical models inhibition of AXL by an antibody-conjugated drug blocked tumour growth (88). TROP2 expression is moderate to high in 86% of ACC, especially in ACC-II (89) and sacituzumab govitecan can be potentially employed in SGC.

Tumor microenvironment resulted as different in two ACC subtypes: in ACC-I more epithelial tumour cells and intratumoural natural killer cells were counted, with a higher expression of Ki67 and B7-H4 and in the stroma more immune cells and cytotoxic T cells were observed; in ACC-II there was a higher density of fibroblasts and myoepithelial p63+ tumour cells (90). To modulate the tumour microenvironment, axitinib and avelumab were used in combination in recurrent/metastatic ACC, providing disease control in most patients without significantly increasing the response rate of historical data with axitinib alone (91).

Non-ACC is a highly heterogenous group of diseases, with many druggable molecular targets. For instance, SDC is an aggressive tumour characterized by overexpression of androgen receptor in 80-90% of cases, HER2 overexpression with higher variability (16-83%), and *PI3KCA*, *HRAS*, and *BRAF* mutations in a lower rate (92). Androgen receptor (AR+) expression supports the use of androgen deprivation therapy and several agents as enzalutamide, abiraterone acetate, apalutamide- have been employed to treat AR+ disease, with favourable outcomes in terms of progression-free survival and overall survival (13). For tumour overexpressing HER2, the use of Herceptin seemed to be reasonable, but in a phase II study, trastuzumab given as a single agent showed a low activity (93). The combination of trastuzumab with docetaxel improved the outcomes with an overall response rate of 70.2% (94). Many other trials are currently ongoing with anti-HER2 agents including new anti HER2 agents (95). The secretory carcinoma carries *ETV6-NTRK3* or *ETV6-RET* fusion in almost all patients and can be treated with entrectinib (96) or larotrectinib (97). Immunotherapy alone cannot provide a significant clinical benefit, especially in ACC (98). Indeed, only high-grade tumours as salivary duct seem to be enriched by PD-L1, compared to ACC, generally defined as immune-excluded tumour (99). Data from recent clinical trials with single agent immunotherapy (e.g. pembrolizumab, nivolumab) are quite disappointing in term of objective response rate (12% at maximum with pembrolizumab) and progression-free survival (median, 4 months (100–103). Results seem to improve with nivolumab and ipilimumab (104). Remarkable, activity of this combination is higher in SDC (25%) compared to non-ACC (16%) and ACC (6%), respectively, suggesting that immunotherapy is promising for very selected histotypes. However, immune-checkpoints have been tested in small patients cohorts with mixed histotypes, further evidence are warranted to deepen the role of immune modulation in SGCs. In addition, there is an urgent need for predictive biomarkers to guide both the therapy and development of effective immuno-oncology combination strategies.

As also recommended in the ESMO/EURACAN/EURACAN guidelines (20), targeted therapy for advanced salivary gland cancers should be based on molecular profiling: indeed, MyPathway phase IIa multiple basket study achieved a 63% of overall response rate with chemotherapy-free regimens matched to specific molecular alteration (105). Clinical trials, however, are warranted in these neglected cancers.

## 9 Conclusion

The current landscape of SGCs is rapidly evolving and impressive advances have been done in the last few years in many fields, including molecular characterization, surgery, radiotherapy, and the development of novel systemic therapies. We have learned that is mandatory to work in research networks to optimize the efforts. Networks are crucial to allow the organization and management of international clinical trials in rare diseases, as SGCs; specific research plans are warranted to support the research in this field.

## Author contributions

LDL: Conceptualization, Supervision, Writing – original draft, Writing – review & editing. RF: Writing – review & editing. LL: Writing – review & editing. MB: Writing – review & editing. LP: Writing – review & editing. DF: Writing – review & editing. GG: Writing – review & editing. DL: Writing – review & editing. PN: Writing – review & editing. VV: Writing – review & editing. MC: Writing – review & editing. BV: Writing – review & editing. GS: Writing – review & editing. PM: Writing – review & editing. IF: Writing – review & editing. DS: Writing – review & editing. AM: Writing – review & editing. EO: Writing – review & editing, Conceptualization, Supervision, Writing – original draft.
